# The Waste from Alcoholic Drinks

**Published:** 1917-05

**Authors:** 


### The Waste From Alcoholic Drinks.

It is said that 600,000,000 bushels of grain is now used in this country in the manufacture of alcoholic drinks, and that people in this and in foreign countries are starving because they cannot get grain to eat. A proposition is being considered to stop the manufacture of alcoholic liquors during the war in order to utilize this grain for food. Heretofore the great argument against intoxicants has been that they unfit men and women for living, make weaklings of them and their children, and unfit them for good citizenship. But if to all the crimes charged against alcoholic drinks is to be added that of starving men, women and children by the hundreds of thousands, by depriving them of needed food, then indeed is alcohol in a bad plight. Heretofore it has been thought that only the families of drunkards went hungry because of liquor, and the voters did not appear to be much concerned for them, but now that all our children may be brought to crying for bread, when it might be had by stopping the manufacture of drink, some kind of prohibition may be expected, at least as a temporary expedient.

Even the saloon keepers of Chicago have come to take this humanitarian view of the situation, and in a recent meeting in that city voted to stand by the President of the United States should he decide that the country should have prohibition during the war.

Should the effect of war prohibition prove as salutary as it is said to be in Russia, there would be but little prospect that liquor drinking would ever again become the curse of this country.

